# Mobile Technologies and Cervical Cancer Screening in Low- and Middle-Income Countries: A Systematic Review

**DOI:** 10.1200/JGO.19.00201

**Published:** 2020-04-17

**Authors:** Dongyu Zhang, Shailesh Advani, Jo Waller, Ana-Paula Cupertino, Alejandra Hurtado-de-Mendoza, Anthony Chicaiza, Peter J. Rohloff, Tomi F. Akinyemiju, Eduardo Gharzouzi, Megan J. Huchko, Joaquin Barnoya, Dejana Braithwaite

**Affiliations:** ^1^ Department of Oncology, Georgetown University School of Medicine, Washington, DC; ^2^ Social Epidemiology Research Unit, Social Behavioral Research Branch, National Human Genome Research Institute, National Institutes of Health, Bethesda, MD; ^3^ Department of Behavioural Science and Health, University College London, London, United Kingdom; ^4^ Latino Cancer Disparities Center, John Theurer Cancer Center, Hackensack, NJ; ^5^ Center for Research in Indigenous Health, Wuqu’ Kawoq, Tecpán, Guatemala; ^6^ Division of Global Health Equity, Brigham & Women’s Hospital, Boston, MA; ^7^ Department of Population Health Sciences, Duke University School of Medicine, Durham, NC; ^8^ Integra Cancer Institute, Guatemala City, Guatemala; ^9^ Duke Global Health Institute, Duke University, Durham, NC; ^10^ Unidad de Cirugia Cardiovascular de Guatemala, Guatemala City, Guatemala

## Abstract

**PURPOSE:**

Cervical cancer screening is not well implemented in many low- and middle-income countries (LMICs). Mobile health (mHealth) refers to utilization of mobile technologies in health promotion and disease management. We aimed to qualitatively synthesize published articles reporting the impact of mHealth on cervical cancer screening–related health behaviors.

**METHODS:**

Three reviewers independently reviewed articles with the following criteria: the exposure or intervention of interest was mHealth, including messages or educational information sent via mobile telephone or e-mail; the comparison was people not using mHealth technology to receive screening-related information, and studies comparing multiple different mHealth interventional strategies were also eligible; the primary outcome was cervical cancer screening uptake, and secondary outcomes included awareness, intention, and knowledge of screening; appropriate research designs included randomized controlled trials and quasi-experimental or observational research; and the study was conducted in an LMIC.

**RESULTS:**

Of the 8 selected studies, 5 treated mobile telephone or message reminders as the exposure or intervention, and 3 compared the effects of different messages on screening uptake. The outcomes were diverse, including screening uptake (n = 4); health beliefs regarding the Papanicolaou (Pap) test (n = 1); knowledge of, attitude toward, and adherence to colpocytologic examination (n = 1); interest in receiving messages about Pap test results or appointment (n = 1); and return for Pap test reports (n = 1).

**CONCLUSION:**

Overall, our systematic review suggests that mobile technologies, particularly telephone reminders or messages, lead to increased Pap test uptake; additional work is needed to unequivocally verify whether mhealth interventions can improve knowledge regarding cervical cancer. Our study will inform mHealth-based interventions for cervical cancer screening promotion in LMICs.

## INTRODUCTION

Cervical cancer is the fourth most common cancer among women worldwide.^1^ Importantly, it remains one of the major gynecologic malignancies threatening quality of life and health status of women in low- and middle-income countries (LMICs).^2^ Yet, cervical cancer is a largely preventable disease.^3^ Specifically, cervical cancer screening with either the Papanicolaou (Pap) or human papillomavirus (HPV) test can identify the presence of precancerous or cancerous cervical cells or high-risk HPV associated with cervical cancer development.^4^ In LMICs, because of the inadequate health service infrastructure and resources, visual inspection with acetic acid (VIA) or Lugol’s iodine (VILI) is widely used to detect early-stage neoplastic lesions.^5,6^ Utilization of these techniques can largely reduce cervical cancer burden. For example, cervical cancer mortality in the United States was significantly reduced after the introduction of the Pap test in the 1950s.^7^ However, similar practices have been less frequently implemented in LMICs than in high-income countries.^8^ Most high-income countries have systematic guidelines for cervical cancer screening; based on 2016 data, approximately two thirds of adult women in the United States underwent a Pap test within the past 3 years.^9^ This is in contrast to practices in LMICs, where implementation of cervical cancer screening is not as widespread as in high-income countries.^10-12^ For example, the 2010 China Chronic Disease and Risk Factor Surveillance System (N = 51,989 women) found that 77% of the sample never underwent the cervical cancer screening.^8^

Mobile health (mHealth) refers to the use of mobile telephones and other wireless technology in health promotion or disease prevention. Overcoming the burden of cervical cancer in LMICs warrants affordable, accessible, and effective technology-based solutions. To our knowledge, no studies have synthesized evidence regarding the effects of mHealth on cervical cancer screening in LMICs. LMICs can benefit greatly from the low-cost, high-reach, high-dissemination capabilities offered by mHealth. As the first step toward developing an mHealth intervention, we set out to systematically review and synthesize evidence from studies examining the association between mHealth and cervical cancer screening in LMICs. Our study has been prospectively registered at PROSPERO (identifier: CRD42018110439).

**CONTEXT**

**Key Objective**
Is it possible to use mobile health (mHealth)–based intervention program to increase awareness, knowledge, and uptake of cervical cancer screening in low- and middle-income countries (LMICs)?
**Knowledge Generated**
Mobile telephone reminders, as compared with other traditional interventions, can increase relevant knowledge and uptake of cervical cancer screening in LMICs. We did not find evidence suggesting differential promoting effects across different types of text messages.
**Relevance**
The outcomes of our study can inform mHealth-based intervention programs in LMICs that aim to promote cervical cancer screening.

## METHODS

### Search Strategy

Four electronic databases (PubMed, EMBASE, Web of Science, and Scopus) were used to search for potentially eligible articles in English until October 10, 2019. Controlled vocabularies (PubMed: MeSH; EMBASE: Emtree) and keywords related to “mobile health,” “phone,” and “cervical cancer screening” were used in the search strategy (Data Supplement). We also searched the reference list of previously published research on similar topics to capture more potentially eligible articles.^13^ A publicly available mHealth Web site^14^ was used to find any additional articles or gray literature relevant to our study. In addition to English articles, D.Z. searched the China Academic Journals Full-Text Database^15^ to obtain potentially eligible articles written in Chinese. D.Z. conducted the search using the keywords “cervical cancer screening”, “mobile phone”, and “mobile”. To obtain potentially eligible articles in Spanish, A.C. searched the Virtual Health Library Regional Portal, a network of Latin American and Caribbean bibliographic databases, for Spanish-language health literature. Spanish-language keywords that were related to “mobile health”, “phone”, and “cervical cancer screening” were used in the search strategy.

### Title and Abstract Screening

For title and abstract screening for English articles, 3 reviewers (D.Z., S.A., and A.C.) independently reviewed titles and abstracts from records identified in electronic databases and decided whether the article should be selected in this process; inconsistent screening decisions regarding inclusion of an article were solved by discussion or by consulting the senior author (D.B.). D.Z. scanned all title and abstract screening records for Chinese articles, whereas A.C. scanned records written in Spanish. Specifically, articles with the following characteristics were included. First, the exposure or intervention of interest was related to mHealth. This included telephone reminder, telephone counseling, text message, smart phone app, e-mail message, and other wireless intervention strategies that conveyed information on cervical cancer prevention, encouraged screening, or provided assistance in screening scheduling. Second, the target population was composed of women eligible for cervical cancer screening, and the comparison group was a nonintervened population or a group of people who did not receive information about cervical cancer screening via mHealth devices or only received such information from traditional media. Traditional media included mailed letters, pamphlets, and newsletters. Studies comparing different types of mHealth approaches were also eligible (eg, telephone call *v* text message). Third, the primary outcome of interest in this systematic review was the uptake of cervical cancer screening (both index and repeat or follow-up screening), and the secondary outcome of interest included awareness, intention, and knowledge of cervical cancer screening. The screening approach included Pap test, HPV test, VIA, or VILI. Fourth, eligible designs were randomized controlled trials (RCTs), quasi-experimental research, or observational studies; reviews and meta-analyses were excluded. Fifth, studies clearly reporting non-LMICs as geographic locations in their titles and abstracts were excluded; studies meeting the first 4 aforementioned criteria without reporting geographic locations were included during the title and abstract screening and further evaluated during the full-text review. We identified LMICs using information provided by the World Bank.^16^ All of the studies meeting the selection criteria in title and abstract screening were included for full-text review.

### Full-Text Review

In the full-text review, reviewers (D.Z., S.A., and A.C.) read whole articles selected during the title and abstract screening to judge whether they should be included for further synthesis. Articles meeting the following criteria were chosen: articles confirmed that study locales were LMICs; the study reported effect measures showing associations between mHealth and cervical cancer screening; and the study had a corresponding full-text article in English, Chinese, or Spanish, and protocols were not included. If duplicated study populations were used in > 1 article, the article with the highest quality (eg, robust study procedures and analytic strategies) was chosen. This process was independently completed by 3 reviewers (D.Z., S.A., and A.C.), and inconsistent decisions were solved by discussion or by consulting the senior author (D.B.). D.Z. read full texts written in Chinese, and A.C. read full texts written in Spanish. We present a flowchart showing the number of studies excluded at each step and summarize the whole selection process and exclusion reasons using a Preferred Reporting Items for Systematic Review and Meta-Analysis (PRISMA) flowchart.^11^

### Data Extraction

We extracted the following characteristics from published articles: name of the first author and publication year; year of data collection; study location; definition of exposure or intervention of interest and comparison; measurement or randomization process of exposure or intervention; sample size; definition and measurement of cervical cancer screening–related outcomes, including uptake, awareness, intention, and knowledge of cervical cancer screening; time period of intervention and follow-up; mean age of women; effect measure and 95% CI of mHealth intervention or exposure; and variables adjusted for in the model. Data extraction was conducted by reviewers independently, and discrepancies between reviewers were resolved by discussion or consulting the senior author (D.B.).

### Qualitative Synthesis and Quality Assessment

A narrative synthesis was conducted to descriptively summarize the main study characteristics (ie, sample size, study locale, average age of participants, and year conducted), definition and measurement of mHealth and cervical cancer screening, measures of association, and major limitations of each study. Specifically, each study was independently assessed for the methodologic strengths and limitations by reviewers (D.Z., S.A., and A.C.), and discrepancies were resolved by discussion or by consulting the senior author (D.B.). We referred to the *Cochrane Handbook for Systematic Reviews of Interventions*^17^ and mainly considered selection bias, measurement error, and analysis strategy when assessing study quality.^7^ For interventional studies (RCTs and quasi-experimental studies), we further evaluated the rationality of randomization and blinding. RevMan 5.3 (Cochrane Collaboration, London, United Kingdom) was used to summarize the risk of bias in intervention studies. We used the modified Newcastle-Ottawa Scale (NOS) to assess the quality of cross-sectional studies.^18^

## RESULTS

### Study Identification and Selection

**FIG 1:**
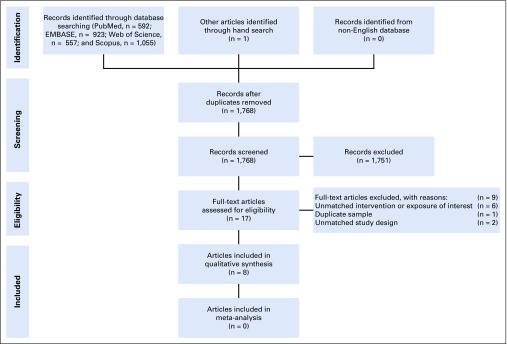
Flowchart of study identification and selection.

Overall, we identified 3,127 records from the electronic databases (PubMed, n = 592; EMBASE, n = 923; Web of Science, n = 557; and Scopus, n = 1,055) and 1 article from other sources. After deduplication, we kept 1,768 articles for title and abstract screening. Of the 17 articles selected from title and abstract screening, 6 were excluded because of unmatched exposure or intervention of interest, 1 was excluded because of duplicate sample use, and 2 were excluded for unmatched study design. This yielded a total of 8 studies^19-26^ included in the systematic review. Because of the large heterogeneity in the definition of mHealth, outcomes of interest, and study design, we did not perform the quantitative synthesis (Fig 1). The PRISMA checklist is provided in the Data Supplement.

### Study Characteristics and Quality

Table 1 lists the study characteristics. The included studies were conducted in different geographic locations and times. One study was conducted in Iran,^21^ 1 was in Tanzania,^26^ 2 were in Malaysia,^19,25^ 2 were in Brazil,^22,24^ and 2 were in South Africa.^20,23^ These studies were all conducted within the past decade, and the time span ranged from 2010 to 2016. Seven of the studies were interventional research studies (2 quasi-experimental studies and 5 RCTs),^19-22,24-26^ and one was a cross-sectional study.^23^ The study samples ranged from 106 to 1,000 participants. Seven studies reported the average age of participants, which ranged from 29 to 42 years.^19-24^ One study^25^ only reported the categorical age distribution, and approximately 40% of people were younger than age 35 years. The exposures and interventions of interest were diverse in these studies. Briefly, 5 studies treated utilization of telephone or message reminder as the exposure or intervention,^19,21,23,25,26^ and 3 studies^20,22,24^ compared effects of different types of text messages on screening. Among the interventional studies,^19-22,24-26^ the length of follow-up ranged from 1 week to 6 months. Overall, there were 5 types of outcomes reported by these studies, and they were as follows: screening uptake (n = 3)^19,20,25,26^; health beliefs about Pap test (n = 1)^21^; knowledge of, attitude toward, and adherence to screening (n = 1)^22^; interest in receiving messages about Pap test report or appointment (n = 1)^23^; and return for Pap test reports (n = 1).^24^

**TABLE 1:**
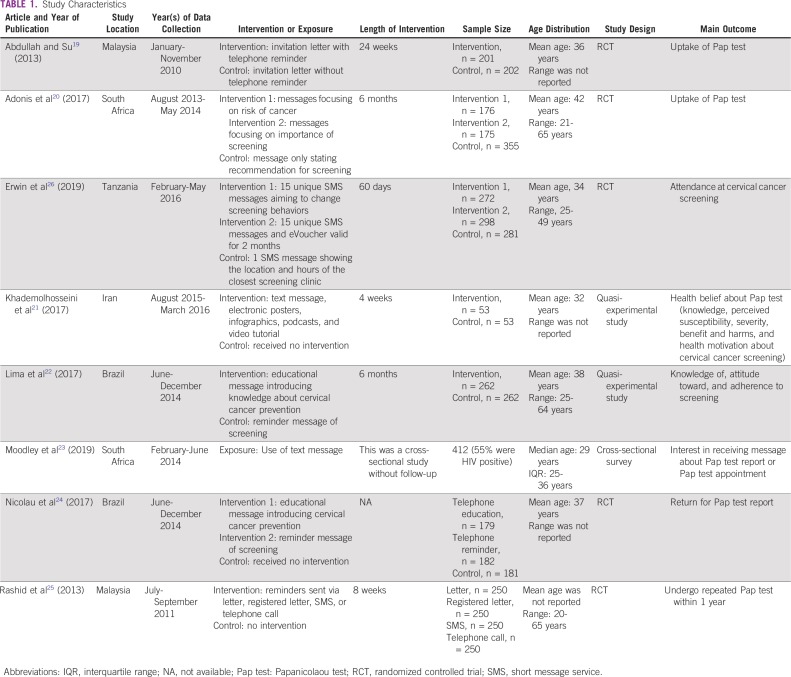
Study Characteristics

Table 2 lists the quantitative outcomes and major limitations in each study. Because of heterogeneous design, study population, intervention or exposure, and definition of outcome, patterns of associations between mHealth and cervical cancer screening seemed to be complex. After a 24-week observation, Abdullah and Su^19^ reported that women receiving invitation letter and telephone reminder were more likely (odds ratio [OR], 2.44; 95% CI, 1.29 to 4.62) to undergo a Pap test compared with women only receiving an invitation letter. Adonis et al^20^ found there was no difference in likelihood of Pap test utilization across people receiving different types of text messages. In particular, as compared with a text message focusing on screening recommendation, messages focusing on the importance of regular screening (OR, 0.57; 95% CI, 0.26 to 1.19) and messages focusing on the risk of cervical cancer (OR, 0.87; 95% CI, 0.47 to 1.66) did not seem to affect Pap test uptake. In a 60-day RCT, Erwin et al^26^ found that sending multiple short message service (SMS) messages with eVoucher (*v* single SMS) significantly increased screening attendance (OR, 4.7; 95% CI, 2.9 to 7.4); the results also showed that participants receiving 15 SMS messages were more likely to attend screening (OR, 3.0; 95% CI, 1.5 to 6.2). A study conducted in Iran applied a comprehensive set of intervention that incorporated text messages, electronic posters, infographics, podcasts, and video tutorials in a quasi-experimental study for 4 weeks.^21^ Consequently, such interventions have been found to positively affect health beliefs and cervical cancer screening knowledge among married women. The intervention versus control group score difference in knowledge was 8.18 points, whereas the corresponding score difference in perceived susceptibility was 4.07. The score differences were 7.78, 2.99, −19.17, and 3.18 for perceived severity, perceived benefits, perceived barriers, and health motivation, respectively. Lima et al^22^ found there was no difference (*P* > .05) in the rates of knowledge of (−3.7%) and attitude toward (−2.0%) colpocytologic examination between women receiving educational versus reminder messages; in addition, although the study found there was an increase in uptake of colpocytologic examination in both groups, the increase was more substantial among women receiving reminder messages (reminder text message, 66.8%; educational text message, 57.5%; rate difference, 9.3%; *P* = .03). A cross-sectional survey reported that women using mobile telephone text messages had a higher interest in receiving appointment reminders via SMS (OR, 14.19; 95% CI, 1.72 to 117.13).^23^ Nicolau et al^24^ reported that an educational message introducing knowledge about cervical cancer (OR, 1.37; 95% CI, 1.22 to 1.54) and a reminder message (OR, 1.40; 95% CI, 1.25 to 1.57) could increase the likelihood of returning to the clinic to receive Pap test results; because their effect measures were quite similar, it suggested there was no difference between their effects on screening-related behaviors. Rashid et al^25^ found that telephone call reminders, as compared with letters, increased the utilization of Pap tests among women who had a history of Pap test in the past year (OR, 2.38; 95% CI, 1.56 to 3.63).

**TABLE 2:**
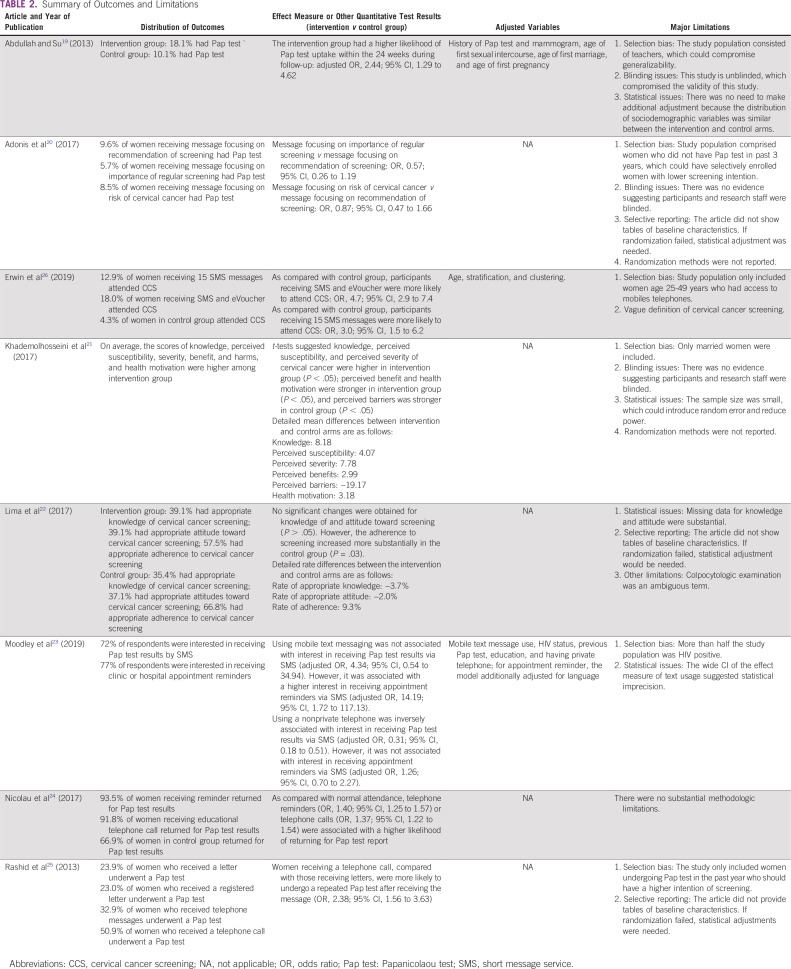
Summary of Outcomes and Limitations

**FIG 2:**
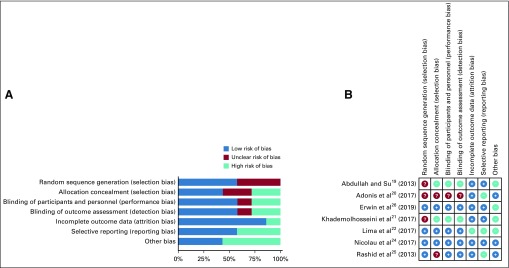
(A) Overall summary of bias of interventional studies. (B) Summary of bias in each interventional study. ?, unclear risk of bias; +, low risk of bias; −, high risk of bias.

The major limitations of included studies involved selection bias, failure to blind, and selective reporting (Table 2). Six of the included studies had selection bias, which compromised the generalizability.^19-21,23,25,26^ Specifically, 1 study only enrolled female teachers,^19^ 1 included women without a Pap test history in the past 3 years,^20^ 1 only enrolled women age 25-49 years with access to mobile telephones,^26^ 1 only included married women,^21^ 1 enrolled a large proportion of HIV-infected women,^23^ and 1 only enrolled women undergoing Pap test with the past year.^25^ These women could be sociodemographically different from the general population; thus, the derived conclusion might be difficult to apply to women at an appropriate age for cervical cancer screening. Three of the 7 interventional studies did not blind their participants or research staff.^19-21^ Although the nature of intervention scenarios made masking difficult, the lack of blinding could still introduce bias in effect measures. For example, women who only received reminder messages might have sought relevant knowledge about cervical cancer if they were aware that their counterparts were receiving professional information about cervical cancer and screening, which might change their screening behaviors. Three interventional studies^20,22,25^ did not report distributions of important sociodemographic variables at baseline, which made it hard for us to judge whether the randomization was successful. In particular, the researchers should have adjusted for other factors if the randomization failed; otherwise, the effect measures could be biased. The risk of bias assessment for interventional studies is presented at Figures 2A and 2B. NOS assessment and potential sources of other bias in interventional studies are provided in the Data Supplement.

## DISCUSSION

To our knowledge, this is the first systematic review that investigates how mHealth can affect cervical cancer screening in LMICs from multiple aspects, including utilization, health beliefs, and interest in receiving relevant appointment information and returning for screening results. Overall, our systematic review suggests that, as compared with traditional communication methods (eg, postal mail), utilization of a telephone reminder or a mobile text message in LMICs can increase uptake of cervical cancer screening. Evidence regarding the effects of mHealth tools on the awareness and perceived threat of cervical cancer is inconclusive and warrants further investigation to delineate optimal implementation strategies in this setting. We did not find robust evidence suggesting that a certain type of text message can have a stronger effect on screening behaviors as compared with others.

Findings from a recently published systematic review that investigated the effects of text messages on cancer screening rates are consistent with our results. Uy et al^27^ synthesized 9 published articles (8 in developed countries and 1 in an LMIC) and concluded that text messaging interventions could increase screening rate for breast (5 studies), cervical (1 study), and colorectal (3 studies) cancer.

This systematic review has some limitations. First, because of the heterogeneity across primary studies and different types of mHealth interventions, we cannot quantitatively synthesize the effect measures, making the intervention effectiveness ambiguous. Second, 3 of the included studies^21,23,24^ did not measure uptake of cervical cancer screening directly, but treated health beliefs about the Pap test, interest in receiving screening appointment, and return for Pap test results as the outcomes of interest. These outcomes can only reflect the awareness of cervical cancer and potential intention of screening and may not guarantee the screening utilization. Third, LMICs consist of countries with differential economic and developmental status. According to the classification of World Bank,^16^ the eight included studies were all conducted in LMICs with better economic situations, which makes our evidence less generalizable to other LMICs with lower economic situations. Fourth, some LMICs may have an organized screening program; however, studies included in this systematic review have insufficient information on how the presence of organized screening programs affected the effectiveness of mHealth interventions targeting cervical cancer screening. It will be important to explore this issue in future research. Furthermore, five of the interventional studies^19-21,24,25^ treated Pap test–related behaviors as the outcomes of interest, and 1 study^22^ used colpocytologic examination as the outcome of interest. However, the cytologic test requires advanced medical equipment and laboratory training, and some low-resource areas do not have such infrastructure, which can reduce accuracy of the cytologic test.^12^ This suggests that future research should examine whether mHealth technology or other wireless devices can affect utilization of HPV testing, which has better screening accuracy in LMICs.^28^

The burden of cervical cancer is higher in LMICs and the corresponding screening rate is lower compared with developed countries,^8,9^ suggesting that an effective and convenient intervention approach is needed in these areas to promote cervical cancer screening. Mobile telephones are much cheaper and portable when compared with laptops, making them easier to use during daily communication. These characteristics demonstrate the potential of such devices to spread knowledge of cervical cancer prevention and promote screening utilization in LMICs. Our results can be informative by providing health practitioners in LMICs with the evidence necessary to establish cost-effective cervical cancer screening promotion programs using mHealth technology. Future studies should explore how mHealth can modify women’s screening behaviors in LMICs with worse economic situations and examine the effectiveness of other mobile devices or technologies, such as telephone apps. Moreover, because numerous cultural and spiritual factors across LMICs influence the uptake of mHealth interventions related to cervical cancer screening, further research is paramount to evaluate their roles.
